# Paraneoplastic Raynaud’s Phenomenon: A Case Report

**DOI:** 10.7759/cureus.99075

**Published:** 2025-12-12

**Authors:** Beatriz Vitó Madureira, Rita Aranha, Rita Soares Costa

**Affiliations:** 1 Internal Medicine, Local Health Unit of Entre Douro e Vouga, Santa Maria da Feira, PRT; 2 Oncology, Local Health Unit of Entre Douro e Vouga, Santa Maria da Feira, PRT

**Keywords:** lung cancer, oncologic complications, paraneoplastic syndromes, raynaud’s phenomenon, vasospasm

## Abstract

Raynaud’s phenomenon is a vasospastic disorder that may occur as a primary benign condition or as a secondary manifestation of systemic disease. We describe the case of a 63-year-old man, with stage IV squamous cell carcinoma of the left lung undergoing palliative chemotherapy, who developed asymmetric and transient discoloration of the distal upper extremities. The patient’s clinical history of active cancer, combined with exclusion of other secondary causes, supported a paraneoplastic etiology. Paraneoplastic Raynaud’s phenomenon should be considered in adults with atypical or new-onset vasospastic symptoms, especially in the context of established malignancy. Early recognition is essential to optimize symptom management and enhance understanding of paraneoplastic vascular syndromes associated with lung cancer.

## Introduction

Raynaud’s phenomenon is a vascular disorder characterized by episodic vasospasm of the digital arteries, typically triggered by cold exposure or emotional stress [1]. While most cases are primary and benign, secondary Raynaud’s phenomenon may signal an underlying systemic disease [2]. Among the less common but clinically relevant causes is paraneoplastic Raynaud’s phenomenon, in which digital vasospasm occurs in association with an underlying malignancy [2-7]. Paraneoplastic Raynaud’s is thought to arise from tumor-related immune dysregulation or circulating vasoactive factors that disrupt normal vascular tone, making it an important clinical clue to occult or evolving malignancy [1,8]. Although it can precede the detection of cancer, it may also arise after the diagnosis has already been established [8,9]. In such cases, treatment of the underlying malignancy often leads to partial or complete resolution of vasospastic symptoms [3,4-7]. We present a case of Raynaud’s phenomenon developing after a confirmed cancer diagnosis and discuss its clinical characteristics and diagnostic considerations.

## Case presentation

A 63-year-old man with stage IV squamous cell carcinoma of the left lung, diagnosed in January 2024 and undergoing palliative chemotherapy, was admitted to the Internal Medicine Department in February for uncontrolled cancer-related pain and a tooth abscess.

**Figure 1:**
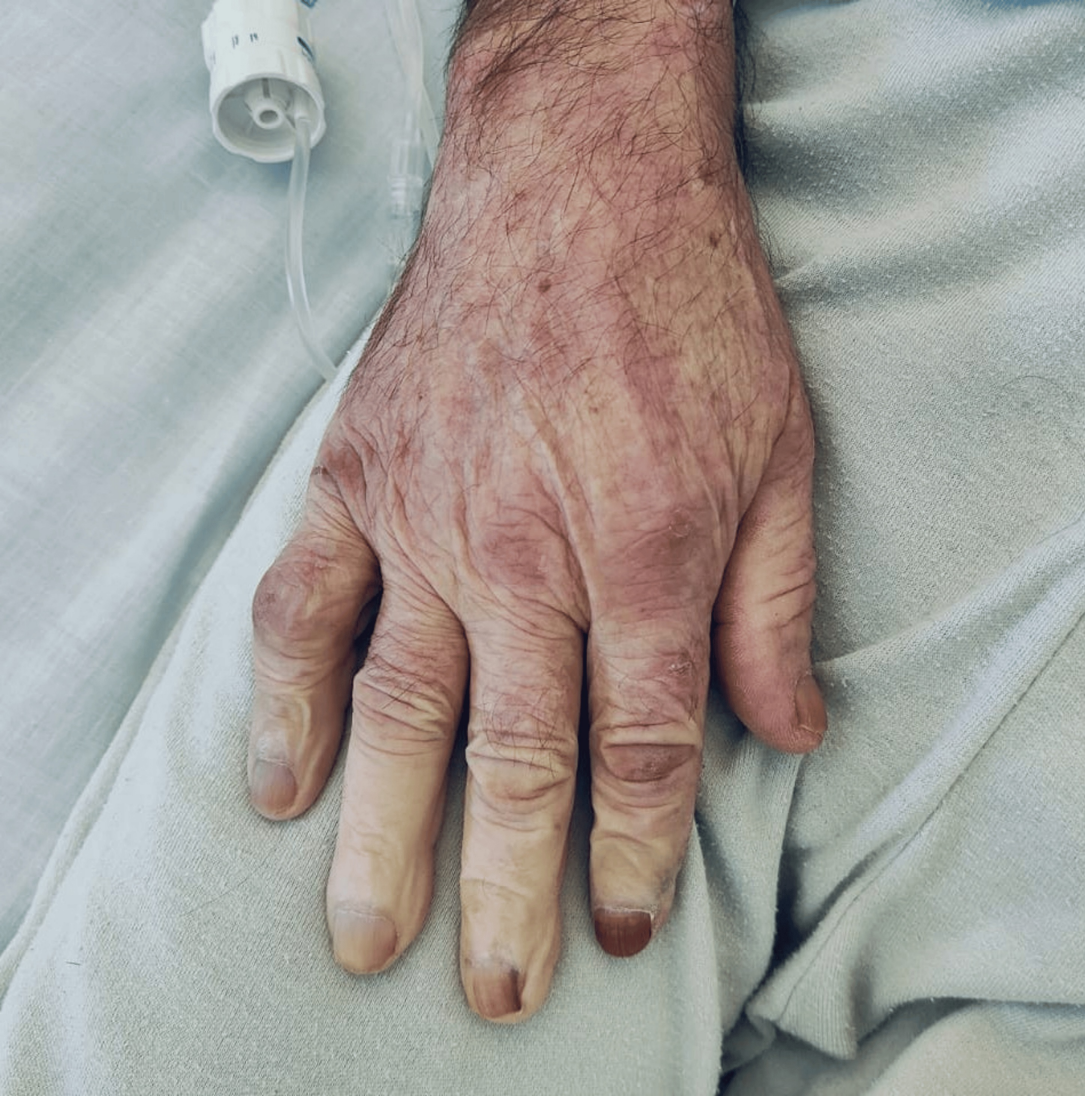
Asymmetrical discoloration of the right hand, involving the distal phalanges The photograph shows well-demarcated areas of digital blanching observed during hospitalization, illustrating the transient vasospasm characteristic of the pallor phase of Raynaud’s phenomenon

During hospitalization, he developed transient episodes of asymmetric discoloration of the distal upper extremities (Figure 1). These episodes were characterized by sudden pallor followed by cyanosis and erythematous reperfusion, each lasting several minutes and accompanied by fingertip pain and numbness. Vital signs and radial pulses were normal; however, the digits demonstrated marked temperature asymmetry with cool, thinned skin. No pitting scars, digital ulcers, or muscle weakness were observed. The patient reported experiencing similar episodes over the previous five months, during outpatient follow-up, with increased frequency in cold environments.

A diagnostic workup was performed to evaluate potential secondary causes (Table 1). Laboratory results showed an elevated erythrocyte sedimentation rate (>120 mm), but negative autoimmune serologies, including antinuclear antibodies, rheumatoid factor, and complement levels. Complete blood count was unremarkable. Serum immunoglobulin levels and protein electrophoresis were within normal limits, excluding plasma cell dyscrasias, cryoglobulinemia-related paraproteins, and other gammopathies. Thyroid function and creatine kinase levels were normal. Acute viral infections, viral hepatitis, and HIV infection were excluded. Urinalysis showed no evidence of renal involvement. Further testing was not pursued because there were no clinical features suggestive of sclerodactyly, digital ulcers, or inflammatory myopathy. Nailfold capillaroscopy could not be performed in a timely manner due to the unavailability at the hospital.

**Table 1:** Laboratory findings during hospitalization in the context of paraneoplastic Raynaud’s phenomenon * indicates values outside the normal reference range. Reference intervals may vary slightly depending on laboratory standards. ** The antibody panel tested included the following: dsDNA, U1-RNP, SSA, SSB, Centromere B, Scl-70, Jo-1, Fibrillarin, RNA Polymerase III, Rib-P, PM-Scl, PCNA, and Mi-2. *** includes testing for Epstein-Barr virus, cytomegalovirus, and varicella-zoster virus The table presents the laboratory results obtained during hospitalization, including complete blood count, coagulation studies, serum biochemistry, immunologic panel, thyroid function tests, viral serologies, and urinalysis. Findings revealed mild anemia and hypoproteinemia, along with elevated erythrocyte sedimentation rate and C-reactive protein, with no additional abnormalities. Overall, the laboratory profile supports the presumed diagnosis of paraneoplastic Raynaud’s phenomenon

Parameter	Result	Unit	Reference range
Hemoglobin	11.7*	g/dL	12.0-16.0
White blood cells	8.3	x10⁹/L	4.0-11.0
Platelets	291	x10⁹/L	150-450
International normalized ratio (INR)	1.1	-	-
Prothrombin time (PT)	12.1	s	Control value: 12.0 s
Activated partial thromboplastin time (aPTT)	30.4	s	Control value: 31.3 s
Urea	20	mg/dL	15-40
Creatinine	0.6	mg/dL	0.6-1.1
Creatine kinase	58	U/L	30-200
Troponin I	1.7	ng/L	≤34.0
Myoglobin	30.4	ng/mL	<140.0
Erythrocyte sedimentation rate	>120*	mm	0-20
C-reactive protein (CRP)	26.6*	mg/L	0.0-5.0
Complement 3	167	mg/dL	82-185
Complement 4	34	mg/dL	15-53
Complement C1q	26	mg/dL	>12
Circulating immune complexes	<0.40	mcg Eq/L	<4.00
Antinuclear antibodies (ANA)**	0.1	U/mL	Negative <0.7
			Positive >1.0
Myeloperoxidase anti-neutrophil cytoplasmic antibodies (MPO-ANCA)	0.20	Ul/mL	Negative <3.5
			Positive >5
Anti-proteinase 3 antibodies (PR3-ANCA)	0.30	Ul/mL	Negative <2
			Doubtful 2-3
			Positive >3
Rheumatoid factor	<20	Ul/mL	<30
Total Proteins	5.80*	g/dL	6.40-8.30
Immunoglobulin A (IgA)	519	mg/dL	101-645
Immunoglobulin M (IgM)	78	mg/dL	22-240
Immunoglobulin G (IgG)	1054	mg/dL	751-1560
Protein electrophoresis	Normal	-	-
Thyroid-stimulating hormone (TSH)	3.76	µUl/mL	0.35-4.94
Free thyroxine (FT4)	10.6	pmol/L	9.0-19.0
Acute viral serology***	Negative	-	-
Viral hepatitis	Negative	-	-
Human immunodeficiency virus (HIV) antibody	Negative	-	-
Urinalysis	Negative for proteinuria, leukocyturia, and erythrocyturia	-	-

Given the recent onset of symptoms occurring shortly after the diagnosis of lung carcinoma, and in the absence of an alternative etiology, paraneoplastic Raynaud’s phenomenon was considered the most likely diagnosis. Following discharge, the patient resumed palliative chemotherapy and began immunotherapy with pembrolizumab. At a three-month follow-up visit, the patient continued to experience similar vasospastic episodes, although they occurred less frequently. Imaging studies demonstrated stability of the underlying malignancy.

## Discussion

Paraneoplastic Raynaud’s phenomenon is an uncommon but clinically significant manifestation associated with several malignancies, most frequently lung, breast, and hematologic cancers [3-7]. In this case, the patient’s recent diagnosis of stage IV squamous cell carcinoma of the lung and the temporal proximity of symptom onset strongly support a paraneoplastic mechanism. While primary Raynaud’s phenomenon is usually benign, secondary forms often present later in life and are frequently asymmetric and more severe [1,2]. The asymmetric and transient digital discoloration observed during hospitalization, combined with a five-month history of cold-induced episodes, is consistent with the expected pattern of secondary Raynaud’s phenomenon.

The diagnostic workup excluded other common causes of secondary Raynaud’s phenomenon [2]. Although the erythrocyte sedimentation rate was markedly elevated, this finding likely reflects the systemic inflammatory response associated with advanced malignancy [10]. This interpretation is supported by negative antinuclear antibodies, normal complement levels, and the absence of clinical features suggestive of autoimmune disease. Endocrine abnormalities, viral infections, and other systemic causes were also ruled out. Although capillaroscopy would have strengthened the diagnostic assessment [2], the temporal association with the cancer diagnosis and the absence of an alternative explanation make paraneoplastic Raynaud’s phenomenon the most plausible diagnosis.

Lung tumors are known to produce a variety of paraneoplastic vascular and neurological syndromes [8,9], and Raynaud’s phenomenon may fall within this spectrum [6-7]. Paraneoplastic manifestations can arise before, during, or after the diagnosis of malignancy, and their course often parallels tumor burden [3,5-9]. In this case, the reduction in symptom frequency over a three-month period is noteworthy, suggesting that even subtle changes in tumor activity may have contributed to the attenuation of vasospastic episodes, despite overall stability of the disease.

Management approaches differ between primary and secondary Raynaud’s phenomenon. Primary Raynaud’s is typically addressed with conservative measures (cold avoidance, smoking cessation, and stress reduction) and supplemented with pharmacologic therapy when needed, most commonly calcium channel blockers [11]. In contrast, secondary Raynaud’s requires both symptomatic treatment and management of the underlying condition [2], which in this case included cancer-directed chemotherapy and immunotherapy.

## Conclusions

Paraneoplastic Raynaud’s phenomenon, although rare, should be considered in adults who present with new-onset, atypical, or asymmetric vasospastic symptoms, particularly when rheumatologic and secondary causes have been excluded. In this case, the temporal association between symptom onset and the diagnosis of advanced lung cancer supports a paraneoplastic mechanism and highlights the need for heightened clinical suspicion in cancer populations. Nonetheless, the diagnosis in this case is limited by the absence of nailfold capillaroscopy and the reliance on temporal correlation rather than definitive biomarkers. Recognizing this entity in patients with an established cancer diagnosis remains important, as new vasospastic symptoms may reflect tumor-related vascular dysregulation and help guide appropriate symptom management. Reporting such cases expands current knowledge of paraneoplastic vascular syndromes and reinforces awareness of their variable clinical presentations.
